# Assessing occupational participation among justice-involved people ‘with a personality disorder’: Quantitative assessments and their properties

**DOI:** 10.1177/03080226241254768

**Published:** 2024-06-03

**Authors:** Catriona Connell, Elizabeth Anne McKay

**Affiliations:** 1 University of Stirling, Stirling, UK; 2 University of Warwick, Coventry, UK; 3 Birmingham and Solihull Mental Health NHS Foundation Trust, Birmingham, UK; 4 Edinburgh Napier University, Edinburgh, UK

**Keywords:** Personality disorder, justice-involved, offender, MOHOST, OPHI-II, occupational participation

## Abstract

**Introduction:**

There is little evidence for what influences occupational participation for justice-involved people ‘with a personality disorder’ living in community contexts, and no validated occupational participation assessments specific to this group. We assessed a sample of justice-involved people ‘with a personality disorder’ to ascertain what influences occupational participation using commonly applied assessments and evaluated their construct validity.

**Method:**

As part of a mixed-methods study, a purposive sample of 18 justice-involved people ‘with a personality disorder’ were scored on the Model of Human Occupational Screening Tool and Occupational Performance History Interview–Version Two scales. Mean scores were calculated per Model of Human Occupational Screening Tool (MOHOST) item and Occupational Performance History Interview–Version Two (OPHI-II) items and scales and compared to published data. Mann–Whitney *U* Tests were used to identify within-sample differences based on demographic characteristics.

**Results:**

Participants had low scores on MOHOST items and OPHI-II items and scales. Differences were identified compared to published data. Within-sample differences were most apparent in comparisons by employment status and ethnicity. The OPHI-II scales did not operate as intended with this population and recommended adjustments impacted its construct validity.

**Conclusion:**

Replication is required with a larger random sample. Integrating these data with qualitative exploration would further elucidate factors influencing occupational participation in this population.

## Introduction

This paper presents quantitative data from a wider mixed-methods study that aimed to identify the factors influencing occupational participation for justice-involved people ‘with a personality disorder’ in community contexts. This paper focuses on the utility of two commonly used tools for assessing occupational participation.

### Background and literature review

Justice-involved people are those who are in receipt of criminal justice sanctions (e.g. prison sentence/community supervision). Justice services internationally have concerns when justice-involved people who present in a way that could indicate a personality disorder are released from custody or receive a community sentence, as this group has increased rates of reoffending (Yu et al., 2012). Justice-involved people with a personality disorder diagnosis experience multiple comorbid health concerns and poorer quality of life compared to justice-involved people without the diagnosis (Black et al., 2010).

Personality disorder is a psychiatric diagnosis characterised by

marked disturbance in personality functioning, which is nearly always associated with considerable personal and social disruption. . . Impairments in self-functioning and/or interpersonal functioning are manifested in maladaptive (e.g., inflexible or poorly regulated) patterns of cognition, emotional experience, emotional expression, and behaviour (World Health Organization, 2018).

The term personality disorder is considered pejorative by many, and those in receipt of the diagnosis experience stigma even within services (Chartonas et al., 2017). Debate about the construct is beyond this paper’s scope, which takes the pragmatic approach of focusing on the population seen in practice.

Approximately 48% of the English probation caseload would meet diagnostic criteria for a personality disorder (Brooker et al., 2012). In England and Wales, the Offender Personality Disorder Pathway (OPDP) was established to work more effectively with men and women considered likely to have a ‘severe personality disorder’ linked to their risk of reoffending (Skett, 2015). People are eligible for OPDP services if a screening assessment (Bui et al., 2016) and clinical review by psychologists determines that they are likely to have a personality disorder. This non-diagnostic approach was adopted to maximise reach to those in need of emotional and interpersonal support, albeit not without controversy, and in partial response to critiques about personality disorder diagnoses (Minoudis and Kane, 2017). The term ‘with a personality disorder’ within inverted commas is adopted in this manuscript to reflect the contested nature of the diagnosis and to acknowledge that participants, like the practice population, were not formally diagnosed.

Occupational therapists are recommended team members in community OPDP services (Her Majesty’s Prison and Probation Service and NHS England, 2020). Their focus on enabling occupational participation is important for justice-involved people ‘with a personality disorder’ because of the challenges the population experience performing valued social roles and the associations between occupational participation and health (World Health Organization, 2002) and reduced risk of reoffending (Olver et al., 2014). The level of difficulty in social roles among those with both justice involvement and a personality disorder diagnosis exceeds those experienced by people with justice involvement or personality disorder diagnosis alone (Hill et al., 2008, 2013).

For effective intervention, it is vital to understand the factors that contribute to occupational participation on an individual and population level. The former is required for personalised intervention tailoring, and the latter for developing and testing interventions that are likely to be effective across individuals and in planning workforce skills.

A systematic review demonstrated that there is limited evidence for what influences of occupational participation for justice-involved people with a personality disorder diagnosis in community contexts. Three studies were identified (one quantitative, one qualitative and one case study), and all were appraised as low quality (Connell et al., 2018). There is a need for research in this area.

In justice contexts, occupational therapists routinely utilise practice-focused assessments underpinned by the Model of Human Occupation (MOHO) to identify factors contributing to occupational participation (Connell, 2016). However, the psychometric properties of these assessments have not been investigated with justice-involved people ‘with a personality disorder’. In this study, occupational participation was assessed with four MOHO assessment scales; one assessed occupational participation at a point in time (Model of Human Occupation Screening Tool; MOHOST; Parkinson et al., 2006), and three considered distal influencing factors (occupational identity, occupational competence, and occupational settings scales within Occupational Performance History Interview–Version Two; OPHI-II; Kielhofner et al., 2004). Construct validity was examined.

## Methods

A purposive sample of justice-involved people ‘with a personality disorder’ participated in the mixed-methods study, with the sampling approach driven by qualitative data requirements. Interview data were used to score participants on the MOHOST (Parkinson et al., 2006) and OPHI-II Scales (Kielhofner et al., 2004). Ethical approval was obtained from the Biomedical Science Research Ethics Committee, University of Warwick (REGO-2016-1822). Additional approvals were obtained from Her Majesty’s Prison and Probation Service (formerly National Offender Management Service) and Birmingham and Solihull Mental Health NHS Foundation Trust.

### Participants

Participants had to be adults (18+ years) living in a community context; be screened into the OPDP (indicative of likely ‘severe personality disorder’) and aware of this; speak English to a level that allowed interview participation; and give informed consent. People were excluded if they presented an unmanageable risk to the researcher as determined by their supervising Offender Manager (supervising probation employee).

### Sampling and recruitment

Stratified purposive sampling (Patton, 2002) was adopted to select participants with diverse characteristics of relevance to offending, personality disorder and occupational participation. Characteristics considered were age, sex, ethnicity, offence type and employment status (proxy of prosocial occupational participation). Women make up only 5% of the National Probation Service (NPS) caseload and thus were oversampled to ensure representation. Using a sampling framework minimised coverage bias, that is, over-coverage of people with certain characteristics in the target population and under-coverage of others (Daniel, 2011). Sampling ceased when saturation was achieved in the qualitative aspect of the overall study (no new findings emerged as interviews were concurrently conducted and analysed).

An NPS administrator applied the sampling framework to the NPS database to identify potential participants and approached their respective Offender Managers to confirm the person met inclusion criteria. Offender Managers received consent from the potential participants for the researcher to contact them. The researcher attended the potential participant’s next routine probation contact to obtain written informed consent. Participants were given time to reflect before continuing with the interview later the same day or at their next routine probation contact. Combining interviews with routine contacts minimised travel costs and participant burden whilst meeting probation requirements for researcher safety. Where a potential participant refused, the NPS administrator identified an alternative potential participant with similar characteristics.

### Data collection

Interviews were conducted at the NPS premises where the participant attended routine appointments. Interviews consisted of two parts: a narrative interview using broad open questions, for example, ‘what is day to-day life like for you?’ which allowed participants to share what was most relevant to them without predetermined topics, and a semi-structured interview utilising OPHI-II to ensure issues relevant to occupational participation were discussed. OPHI-II covers five themes (daily routine, roles, activity choices, the environment and turning points) in relation to the past, present and future. The semi-structured nature provided more guidance to participants but continued to use questions designed to elicit narrative responses. The order of questioning was amended with advice from the Patient and Public Involvement Advisory Group to maximise rapport with the participant before discussing more sensitive topics. Interviewer-rated assessment scales were completed based on the interviews: MOHOST and the three OPHI-II scales (occupational identity, occupational competence, and occupational settings).

#### Model of Human Occupation Screening Tool (MOHOST)

MOHOST measures components of occupational participation at a point in time using a four-point ordinal rating scale on 24 items across six subscales. Subscales have four items each and represent motivation for occupation, pattern of occupation, communication and interaction skills, process (cognitive) skills, motor skills, and the environment. Each point on the scale is assigned a letter. *F* indicates the item facilitates participation, A that it allows participation, I that it inhibits participation and R that it restricts participation. Scores are determined using manual criteria (Parkinson et al., 2006). MOHOST has demonstrated construct, convergent and discriminant validity, and person separation reliability with a sample of people with mental health conditions (Kielhofner et al., 2010) and is sensitive to individual-level change over time in patients in forensic mental health settings (Fan et al., 2016). Although the forensic mental health sample consisted predominantly of people with schizophrenia, many people in forensic services have comorbid personality disorder diagnoses. Therefore, MOHOST was suitable for measuring components of occupational participation in this study.

#### Occupational Performance History Interview–Version 2 (OPHI-II)

The OPHI-II scales measure occupational identity, occupational competence, and occupational settings (Kielhofner et al., 2004). These three concepts are theorised to underpin the occupational adaptation process and, therefore, influence occupational participation. The occupational identity scale consists of 11 items that measure ‘the degree to which a person has internalised a positive occupational identity’ (p.5). The occupational competence scale consists of nine items that measure the ‘degree to which one is able to sustain a pattern of occupational participation that reflects one’s occupational identity’ (p. 5). The occupational settings scale consists of nine items that measure ‘the impact of the environment on the client’s occupational life’ (p. 5). On the OPHI-II scales, the interviewer scores items on a four-point ordinal scale using manual criteria. People score 4 when exceptionally competent, 3 for good/appropriate/satisfactory, 2 for some problems and 1 for extreme problems. Ordinal data from the item scores is converted to interval data to produce an overall score for each scale. Where item scores do not fit the expected pattern, a rater has two options: to conclude the measure is not working as intended and omit the scoring system; or to exclude scores that deviate from expectations to produce an adjusted score (Kielhofner et al., 2005). In a study of the psychometric properties of the OPHI-II scales, including people with mental health diagnoses, all scales demonstrated person and item separation reliability, and construct and discriminant validity. The scoring assessors in the validation study were mostly self-trained (by reading the manual) or attended a lecture, reflecting the realities of practice training (Kielhofner et al., 2001). The inclusion of people with mental disorders in validating its psychometric properties, its strong theoretical basis, and its usability based on self-training indicate the OPHI-II scales were suitable for this study.

### Data analysis

#### MOHOST item scores

MOHOST item scores for each participant were converted to numeric scores where 1 represented ‘restricts participation’ and 4 represented ‘facilitates participation’. Mean scores on MOHOST items were used to identify trends in areas of strengths and limitations across participants. Mean scores above 3.5 were taken to indicate an area was a strength, based on a pragmatic logic that this would equate to an item ‘facilitating’ occupation (rather than just allowing it) when applied to an individual. Mean scores below 2.5 were considered limitations, as when applied to an individual, this would indicate an item restricted or inhibited occupational participation.

#### MOHOST subscale scores in comparison to other samples

Mean subscale scores per participant were produced by taking a mean of the four item scores in each subscale (i.e. mean of the four items describing motivation for occupation). Although inexact to combine ordinal data this way, it allowed comparison to a sample of United Kingdom mental health (UKMH) service users (Lee et al., 2011). Lee et al. (2011) also present data by ‘care cluster’, a way of categorising levels and types of mental health needs in the United Kingdom (UK) (NHS England, 2016). Mean subscale scores from this study were compared to Lee et al.’s full sample and the scores for people in cluster eight (‘non-psychotic chaotic and challenging disorders’; NPCC), which would typically include those diagnosed with a personality disorder. Published data did not allow statistical comparison; thus, to determine meaningful differences between the study sample and published data, two approaches were combined. Firstly, a meaningful difference was determined to exist where published means for the UKMH or NPCC samples fell outside the 95% confidence interval of the mean scores in the sample in this study. Secondly, whether the difference was clinically important was considered. The minimal clinically important difference (MCID) is the smallest change in score that can be recognised in reality by the service user or clinician in the concept being measured (Jaeschke et al., 1989). This is based on the understanding that a change in score may be measurable but not produce observable change. For example, a 0.1 difference in means for motivation could suggest that one group was more enabled by their levels of motivation, but in reality, such a small difference could mean that both groups had restrictive levels of motivation. Using a pragmatic approach, an MCID was determined as a difference in score of 0.5, as this would reflect the score needed for someone to move between levels on the ordinal scale on an individual basis (e.g. from inhibiting to allowing occupational participation) and would thus be an observable difference.

#### OPHI-II item scores

Mean scores on items in each of the three OPHI-II scales were calculated and used to identify trends within the sample in areas of strengths and limitations using the same approach as with MOHOST items.

#### OPHI-II scale scores in comparison to other samples

OPHI-II scale scores were compared to published scores for people with physical disability and no diagnosis (Kielhofner et al., 2001). Statistical comparison was not possible from the published data. MCIDs have not been established for the OPHI-II scales, and similar pragmatic assumptions about this were not possible. Therefore, a meaningful difference in scores was considered. This was determined to be where the published scores fell outside the 95% confidence interval for the mean scale scores of this study. Where item scores do not fit expected patterns, the OPHI-II manual advises excluding these scores when determining the scale score to produce an adjusted score (Kielhofner et al., 2004). Comparisons were run with unadjusted and adjusted scores.

#### Within sample sub-group comparisons

Within the sample of justice-involved people ‘with a personality disorder’, MOHOST item, subscale and OPHI-II item and scale scores were compared statistically between groups based on sex (men vs women), age (<35 vs 35+), ethnicity (white vs minority ethnicities), offence type (violent vs sexual) and employment status (employed vs unemployed). Rationales for dichotomising data in these ways are presented in the Supplementary File. MOHOST items and subscales and OPHI-II items were assumed to be representative of interval data, that is, that there is an underlying continuum. Distributions were not normal across items or scales, and given our small sample size, statistical comparisons were conducted using non-parametric Mann–Whitney *U* tests. Significance level was set at *p* < 0.05. Analyses were run in SPSS 24 (IBM Corp, 2016).

## Results

### Participants

Eighteen participants with diverse demographic characteristics were interviewed. See Table 1. Results should be considered with this small sample size in mind.

**Table 1.** Participant demographics.

Participant demographics	Men *N* (%)	Women *N* (%)	Total *N* (%)
Age			
Under 35	8 (62)	1 (20)	9 (70)
35+	5 (38)	4 (80)	7 (39)
Ethnicity			
White	10 (77)	3 (60)	13 (72)
Ethnic minority	3 (23)	32 (40)	5 (28)
Offence type			
Violent	9 (69)	4 (80)	13 (72)
Sexual	4 (31)	1 (20)	5 (28)
Employment status			
Employed	4 (31)	1 (20)	5 (28)
Unemployed	9 (69)	4 (80)	13 (72)

### MOHOST item scores

**Figure 1.**
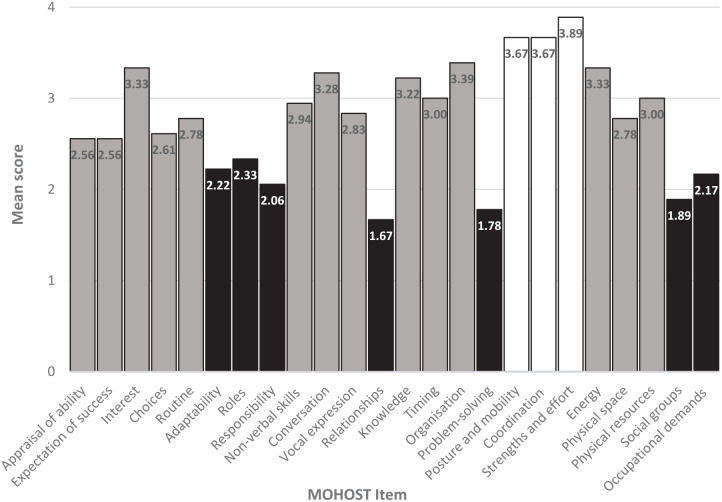
Mean MOHOST item scores. MOHOST: Model of Human Occupational Screening Tool.

Figure 1 shows the mean MOHOST item scores. Items that scored below 2.5 on average, indicating an impairment, were: adaptability, roles, responsibility, problem solving, social groups, and occupational demand. Items scoring above 3.5 on average, indicating a strength, were posture and mobility, coordination and strength and effort.

### MOHOST subscales in comparison to published samples

Compared to the UKMH sample, the study sample of justice-involved people ‘with a personality disorder’ (henceforth the study sample) had lower mean age (mean 36.2 years, s.d. 10.6 vs mean 58.8 years, s.d. 22.1), contained a higher proportion of men (72.2% vs 43.7%) and a higher proportion of people in employment (27.8% vs 7.9%). The mean motor skills subscale score was the only comparison that demonstrated a meaningful difference (where the published UKMH mean fell outside the 95% confidence interval for the mean in the study sample) and MCID (study mean = 3.64, CI = 3.38–3.90; UKMH mean 3.09). On average, the study sample had higher motivation, process skillls and motor skills but a less supportive environment than the UKMH sample.

Compared to the NPCC sample, the study sample was, on average, younger (36.2 years, s.d. 10.6 vs 46.0 years, s.d. not reported) with a higher proportion of men (72.2% vs 43.5%). The mean communication and interaction skills subscale score was the only comparison to show a meaningful difference and MCID (study mean = 2.68, CI 2.35–3.01; NPCC mean = 3.25). Several published mean subscale scores fell outside the 95% confidence interval for the study sample, but did not differ by 0.5 or more to reach a MCID. All comparisons and confidence intervals are reported in Table S1 in the Supplementary File.

### OPHI-II item scores

**Figure 2.**
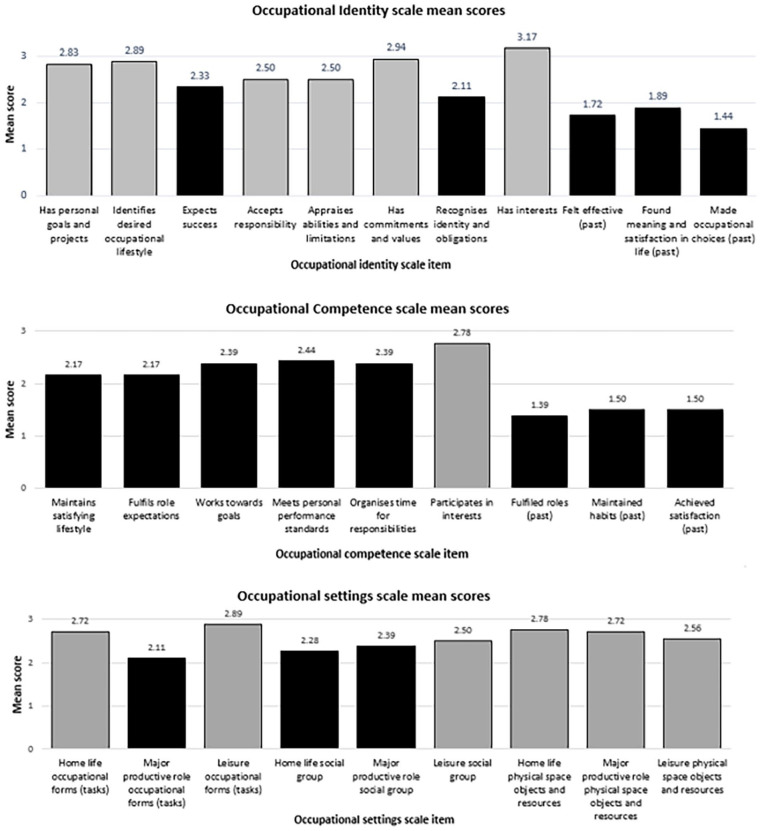
Mean OPHI-II scale item scores. OPHI-II: Occupational Performance History Interview–Version Two.

Figure 2 shows mean item scores on the occupational identity, competence, and settings scales. No items had a mean score above 3.5, indicating a strength. Limitations (coloured black) were identified in 5 of 11 items on the occupational identity scale, eight of nine on the occupational competence scale and three of nine on the occupational settings scale. All scores relating to past occupational identity and occupational competence were rated as limitations.

### OPHI-II scales

As inferring MCIDs is not possible on the OPHI-II scales, only a meaningful difference was calculated (where the mean from the published sample fell outside the 95% confidence interval from the study sample). Most participants’ scores deviated from expectations set out in the manual on the occupational identity and competence scales, that is, scoring lower rather than higher on items considered easier. Deviations were on ratings of past occupational identity and past occupational competence. The influence of the past is theoretically important and was confirmed to be an influencing factor in qualitative aspects of the study (Connell et al., 2019), thus analyses were run with and without the recommended adjustment of excluding scores that differ from expectations.

Before adjustments, a meaningful difference was identified between the study sample and people with physical disability and people with no diagnosis on the occupational identity scale (study mean = 42.61, CI = 38.03–47.19; physical disability = 58.59; no diagnosis = 60.30) and occupational competence scale (study mean = 40.33, CI = 34.47–46.19; physical disability = 46.94; no diagnosis = 50.14). Following adjustments, there was no longer a meaningful difference on occupational competence scale scores. The meaningful difference remained with both comparison samples on the occupational identity scale. Occupational settings scale scores did not require adjustment. The study sample had a lower mean score, but only comparison with people with no diagnoses reached a meaningful difference (study mean = 47.44, CI = 41.79–53.09, physical disability = 49.59, no diagnosis = 55.31). See Table S2 in the Supplementary File for additional detail.

### Statistically significant differences between groups within the study sample

Because numbers within groups are small, these results are exploratory. Statistically significant differences were identified on MOHOST item scores by sex for non-verbal skills (*p* = 0.041) and timing (*p* = 0.046), with women on average scoring higher than men. On the OPHI-II items, the only statistically significant difference was on the score for ‘felt effective in the past’, where women scored lower than men on average (*p* = 0.046). There were no differences on the MOHOST subscales or OPHI-II scales by sex.

Statistically significant differences were identified across the scales based on ethnicity and employment status. These are presented in Table 2. Minority ethnicity participants scored higher on average than white participants on MOHOST items (routine, adaptability, communication and interaction) and OPHI-II items (has personal goals and projects, major productive role occupational forms, major productive role social groups and leisure physical space, objects and resources). Comparison based on ethnicity was the only comparison to show a difference on the occupational settings scale. Participants in employment scored higher on average than unemployed participants on MOHOST items (responsibilities, problem solving), the MOHOST habituation subscale, eight OPHI-II items (has personal goals and projects, made occupational choices in the past, maintains a satisfying lifestyle, fulfils role expectations, works towards goals, organises time for responsibilities, participates in interests, fulfilled roles in the past) and adjusted and unadjusted OPHI-II scales.

**Table 2.** Statistically significant differences identified by ethnicity and employment status.

Comparison	*p* Value
**Ethnicity**	
MOHOST items	
Routine	0.046
Adaptability	0.026
Communication and interaction	0.046
OPHI-II items	
Has personal goals and projects	0.019
Major productive role occupational forms	0.035
Major productive role social groups	0.026
Leisure physical space, objects and resources	0.026
OPHI-II scales	
Occupational settings	0.026
**Employment status**	
MOHOST items	
Responsibilities	0.046
Problem solving	0.046
MOHOST subscales	
Habituation	0.046
OPHI-II items	
Has personal goals and projects	0.019
Made occupational choices in the past	0.019
Maintains a satisfying lifestyle	0.014
Fulfils role expectations	0.026
Works towards goals	0.026
Organises time for responsibilities	0.003
Participates in interests	0.035
Fulfilled roles in the past	0.026
OPHI-II Scales	
Unadjusted occupational identity	0.003
Adjusted occupational identity	0.007
Unadjusted occupational competence	0.001
Adjusted occupational competence	0.002

MOHOST: Model of Human Occupational Screening Tool; OPHI-II: Occupational Performance History Interview–Version Two.

No statistically significant differences were found between participants based on offence type or age. Full results are presented in Supplementary File Tables S3–S7.

## Discussion

MOHOST and OPHI-II scales were used to identify factors that influence occupational participation with 18 justice-involved people ‘with a personality disorder’. Trends in assessment items were identified within the study sample, and meaningful differences were identified on MOHOST subscales and OPHI-II scale scores compared with published data from other samples. Statistically significant differences were identified within the study sample by sex, ethnicity, and employment status. There were issues with the construct validity of the OPHI-II scales with the study sample. The sample size is small, and all results should be considered carefully.

Justice-involved people ‘with a personality disorder’ presented with a pattern of scores on MOHOST items whereby limitations reflected diagnostic criteria for personality disorder (World Health Organization, 2018). Strengths were seen in motor skills, which are unrelated to personality disorder diagnosis. Compared to a UKMH sample, the only subscale to show a difference was ‘motor skills’, which likely reflects the younger average age of the study sample and inclusion of people with dementia in the UKMH sample. Compared to the NPCC sample, mean score was lower in the study sample on the MOHOST communication and interaction subscale, which includes the item ‘relationships’. Difficulties in relationships are central to a personality disorder diagnosis (World Health Organization, 2018), but this result suggests adding an offending history may cause further difficulties in communication and interaction that impact occupational participation. One heightened difficulty may be a stigma experienced due to justice involvement. Former forensic mental health inpatients with psychotic disorders described experiencing stigma and rejection even within the mental health community, which contributed to hesitance in participating in social relationships (Alred, 2018). Further, among people released from prison in the USA, anticipating personally experiencing stigma predicted lower community functioning (Moore et al., 2016).

The UKMH and NPCC samples reflect populations that face sizeable challenges to occupational participation; thus, areas of difficulty among justice-involved people ‘with a personality disorder’ may have been more clearly identified if scores were contrasted with people with no diagnosis or normative data. Publishing normative data would be a beneficial line of future research. Though the comparison method used in this study was inexact, this is the first occupational therapy study, to our knowledge, to conduct any measurement and comparison including justice-involved people ‘with a personality disorder’, and comparisons were more robust than simply comparing means.

Compared to people with no diagnosis, justice-involved people ‘with a personality disorder’ scored lower on average on all OPHI-II scales. This was considered a meaningful difference for all comparisons except adjusted occupational competence. When compared to people with a physical disability, scores were lower on all comparisons except adjusted occupational competence and occupational settings. These results suggest that the barriers to occupational participation for justice-involved people ‘with a personality disorder’ may be greater than those experienced by people with physical disabilities. The merits and problems of adjusting scores are discussed below.

Item scores on the OPHI-II scales in this sample showed marked impairment on items related to past occupational identity and past occupational competence. These items are reported to be easiest to score well on (Kielhofner et al., 2001), likely because the OPHI-II was developed on the assumption that people usually live a satisfying life before a disruption (illness or injury) impacts their occupational participation (Kielhofner et al., 2004). Most participants described past adversity, traumatic experiences (e.g. being a victim of abuse), difficulty achieving normal developmental roles and past participation in socially unacceptable occupations. According to manual criteria, these should result in lower scores and thus many participants’ scores did not fit expected patterns. These are common experiences among justice-involved people and people with personality disorder diagnosis (Singleton et al., 1998; Afifi et al., 2011). The scales used in this study, and the MOHO on which they are based, conceive of occupational participation in its prosocial form. Critique of occupation and occupational participation as always positive and health promoting, and the bias towards socially valued (as opposed to ‘antisocial’) occupations, is increasing (e.g. Twinley, 2020). Evidence demonstrating these limitations is clear. For example, people ‘with a personality disorder’ participate in occupations considered socially unacceptable for the same reasons people participate in prosocial occupations (e.g. Connell et al., 2019; Potvin et al., 2019). Given the aim of occupational therapists is to enable occupational participation in its prosocial form, the prosocial focus of MOHO and the MOHO assessment tools are not necessarily problematic. Nonetheless, addressing this mis-fitting pattern must be carefully considered.

Analyses excluding non-fitting item scores produced higher occupational identity and occupational competence scale scores. The adjustment is problematic for two reasons. Firstly, score adjustments may result in an overestimate of occupational competence and occupational identity and, thus, perceptions of occupational participation. Secondly, adjusting scores by removing items rating past experiences would remove assessment components that consider the influence of the past on current occupational participation. Adjusting the OPHI-II scale scores in this way, therefore, undermines construct validity of the tool, that is, the extent to which the scales measure their intended constructs (occupational identity and occupational competence are shaped over time by occupational participation and vice versa). Past occupational participation impacted markedly on participants’ present occupational participation, as it does with other populations (whether socially valued or not), and is this vital to consider. How this was experienced is discussed with the qualitative findings (Connell et al., 2019).

An alternative conclusion is that the OPHI-II is not working as intended for this population. However, this may be premature. Past or current participation in ‘antisocial’ occupations are scored low but these developmental trajectories need to be considered for their impact on *prosocial* occupational participation in the present. Without their inclusion, occupational participation among justice-involved people ‘with a personality disorder’ cannot be fully explained. Therefore, it appears that the tool may work as intended by demonstrating the marked impact of past occupational participation on present *prosocial* occupational participation. The assumptions underpinning the expected pattern (a previously successful life disrupted by acquired disability or illness) and the suggested removal of items that do not fit may require reconsideration when applying OPHI-II assessment scoring to justice-involved people ‘with a personality disorder’ and potentially other populations who have not followed Western developmental norms.

In examining differences within the sample, small numbers must be recognised and thus, results are considered preliminary. Most statistically significant differences found were in the employment comparison. This was the only comparison to identify statistically significant differences in occupational identity and occupational competence. Employment status was used as a proxy for successful occupational participation, so higher scores among those in employment would be expected. This suggests the OPHI-II scales may be sensitive enough to identify levels of occupational participation in justice-involved people ‘with a personality disorder’. The second largest number of statistically significant differences were found between participants who were white and those from a UK minority ethnicity. This was the only comparison to identify a difference on the occupational settings scale, which favoured participants from a UK minority ethnicity. This suggests that being from a minority ethnicity in the UK may result in greater access to environments that facilitate occupational participation, such as those with less stigma. Alternatively, restrictive environments may have less effect on people from a minority ethnicity. This may seem counterintuitive, but it has been observed elsewhere. Despite equal levels of *anticipated* stigma between white and African American people released from prison, impaired community participation was only predicted in white participants (Moore et al., 2016). The authors hypothesised that African American participants had likely experienced stigma and race discrimination previously and were thus better psychologically prepared and protected against it, enabling them to participate in the community more successfully (Moore et al., 2016). Given the small sample size in this study, exploration in a larger sample is needed to definitively determine environmental differences experienced by people from different ethnic/racialised backgrounds, if and how this relates to occupational participation, and whether a supportive environment mediates the relationship between anticipated stigma and occupational participation among justice-involved people ‘with a personality disorder’.

Few statistically significant differences were identified by sex and will need to be confirmed in a larger sample to conclude the assessments work equally well for men and women. There is evidence for symptomatic recovery from personality disorder over time (Black, 2015). However, impairments in occupational participation often remain (Zanarini et al., 2012), potentially explaining why no differences were identified by age. People convicted of a sexual offence experience stigma even within offender populations, which is associated with avoidance of community participation (Mingus and Burchfield, 2012). However, no statistically significant differences in scores were identified by offence type.

## Strength and limitations

This is the first research to assess occupational participation with justice-involved people ‘with a personality disorder’ in the community. Results are preliminary owing to the small non-random sample of people without a formal diagnosis, pragmatic comparison method and limitations in the assessments’ psychometric properties. Nonetheless, trends and differences were identified using conservative comparisons in a realistic clinical population, which warrant further examination with larger samples.

Having a single assessment rater ensures consistency but does not guarantee an absence of error bias. Assessments were scored based on interview content and thus may be influenced by how participants described, and the rater interpreted, their experiences (e.g. where a participant wished to appear more able than they were). However, the assessments utilise standardised scoring criteria, and interviews did not involve participants directly describing their strengths and limitations but describing their experiences. Thus, bias introduced is thought to be limited. Further, the assessments have been demonstrated to be reliable and valid without specific training (Kielhofner et al., 2001, 2010), are routinely used in clinical practice in this way, and the rater had experience assessing justice-involved people ‘with a personality disorder’ in practice.

## Conclusion

Factors influencing occupational participation for justice-involved people ‘with a personality disorder’ in the community can be identified using MOHOST and OPHI-II scales. Areas of impairment were consistent with diagnostic criteria for personality disorders and consistent with the impact of having a history of difficulty in occupational participation. The OPHI-II scales often did not produce an expected pattern of scores for occupational identity and occupational competence. These results highlight problems with the assumptions of the OPHI-II, namely that people have previously achieved a socially valued occupational identity and the occupational competence to support it. Unadjusted OPHI-II scale scores, including past impairments, may more accurately represent the occupational participation levels of justice-involved people ‘with a personality disorder’ than excluding unexpected scoring. It would also maintain OPHI-II scale construct validity. Statistically significant differences in scores were identified when subgroups based on sex, ethnicity and employment status, but not age or offence type. However, the small sample size indicates these are tentative and should be investigated in a larger sample.

### Implications for practice

- Occupational therapists should consider occupational participation at a point in time and the historical influences that shape this when working with justice-involved people ‘with a personality disorder’. Due to different life experiences compared to those with whom assessments were developed, scoring should be used indicatively only.

### Implications for research

- MOHO and associated psychometric measures warrant further examination with populations whose past occupational participation deviates from socially valued and expected norms, particularly as this relates to occupational identity and occupational competence.
- Qualitative exploration of occupational participation would help identify areas relevant to justice-involved people ‘with a personality disorder’ that are less clearly explained by MOHO and are not captured in assessments.
- Normative data and minimal clinically important differences should be developed for MOHOST and the OPHI-II scales to allow statistical and clinically meaningful comparisons.

**Key findings**

- Factors influencing occupational participation for justice-involved people ‘with a personality disorder’ can be identified using MOHOST and OPHI-II scales.
- Limitations in the psychometric properties of OPHI-II indicate scale scores should be considered carefully.

**What the study has added**

This is the first study to quantitatively describe occupational participation among justice-involved people ‘with a personality disorder’ in the community and evaluate the psychometric properties of existing assessments with this population.

## Supplemental Material

sj-docx-1-bjo-10.1177_03080226241254768 – Supplemental material for Assessing occupational participation among justice-involved people ‘with a personality disorder’: Quantitative assessments and their properties Supplemental material, sj-docx-1-bjo-10.1177_03080226241254768 for Assessing occupational participation among justice-involved people ‘with a personality disorder’: Quantitative assessments and their properties by Catriona Connell and Elizabeth Anne McKay in British Journal of Occupational Therapy

## References

[bibr1-03080226241254768] Afifi TO Mather A Boman J , et al. (2011) Childhood adversity and personality disorders: Results from a nationally representative population-based study. Journal of Psychiatric Research 45: 814–822. 21146190 10.1016/j.jpsychires.2010.11.008

[bibr2-03080226241254768] Alred D (2018) Service user perspectives of preparation for living in the community following discharge from a secure mental health unit. PhD Thesis. Brighton: University of Brighton.

[bibr3-03080226241254768] Black DW (2015) The natural history of antisocial personality disorder. Canadian Journal of Psychiatry 60: 309–314. 26175389 10.1177/070674371506000703 PMC4500180

[bibr4-03080226241254768] Black DW Gunter T Loveless P , et al. (2010) Antisocial personality disorder in incarcerated offenders: Psychiatric comorbidity and quality of life. Annals of Clinical Psychiatry 22: 113–120. 20445838

[bibr5-03080226241254768] Brooker C Sirdifield C Blizard R , et al. (2012) Probation and mental illness. Journal of Forensic Psychiatry and Psychology 23: 522–537.

[bibr6-03080226241254768] Bui L Ullrich S Coid JW (2016) Screening for mental disorder using the UK national offender assessment system. Journal of Forensic Psychiatry & Psychology 27: 786–801.

[bibr7-03080226241254768] Chartonas D Kyratsous M Dracass S , et al. (2017) Personality disorder: Still the patients psychiatrists dislike? BJPsych Bulletin 41: 12–17. 28184311 10.1192/pb.bp.115.052456 PMC5288087

[bibr8-03080226241254768] Connell C (2016) Forensic occupational therapy to reduce risk of reoffending: A survey of practice in the United Kingdom. Journal of Forensic Psychiatry and Psychology 27: 907–928.

[bibr9-03080226241254768] Connell C Furtado V McKay EA , et al. (2018) What influences social outcomes among offenders with personality disorder: A systematic review. Criminal Behaviour and Mental Health 28: 390–396. 29920809 10.1002/cbm.2082

[bibr10-03080226241254768] Connell C McKay EA Furtado V , et al. (2019) People with severe problematic personality traits and offending histories: What influences occupational participation? European Psychiatry 60: 14–19. 31100608 10.1016/j.eurpsy.2019.05.002

[bibr11-03080226241254768] Daniel J (2011) Sampling Essentials: Practical Guidelines for Making Sampling Choices. London: SAGE.

[bibr12-03080226241254768] Fan C-W Morley M Garnham M , et al. (2016) Examining changes in occupational participation in forensic patients using the Model of Human Occupation Screening Tool. British Journal of Occupational Therapy 79: 727–733.

[bibr13-03080226241254768] Her Majesty’s Prison and Probation Service and NHS England (2020) Intensive Intervention and Risk Management Service specification. London: Her Majesty’s Prison and Probation Service & NHS England.

[bibr14-03080226241254768] Hill J Nathan R Shattock L (2013) Report of a pilot randomized controlled trial of an intensive psychosocial intervention for high risk personality disordered offenders (the ‘Resettle’ programme). University of Manchester. Unpublished Report.

[bibr15-03080226241254768] Hill J Pilkonis P Morse J , et al. (2008) Social domain dysfunction and disorganization in borderline personality disorder. Psychological Medicine 38: 135–146. 17892627 10.1017/S0033291707001626 PMC2828321

[bibr16-03080226241254768] IBM Corp. (2016). IBM SPSS Statistics for Windows, Version 24.0. Armonk, NY: IBM Corp.

[bibr17-03080226241254768] Jaeschke R Singer J Guyatt GH (1989) Measurement of health status. Ascertaining the minimal clinically important difference. Contemporary Clinical Trials 10: 407–415. 10.1016/0197-2456(89)90005-6 2691207

[bibr18-03080226241254768] Kielhofner G Dobria L Forsyth K , et al. (2005) The Construction of keyforms for obtaining instantaneous measures from the Occupational Performance History Interview rating scales. OTJR: Occupation, Participation and Health 25: 23–32.

[bibr19-03080226241254768] Kielhofner G Fan C-W Morley M , et al. (2010) A psychometric study of the Model of Human Occupation Screening Tool (MOHOST). Hong Kong Journal of Occupational Therapy 20: 63–70.

[bibr20-03080226241254768] Kielhofner G Mallinson T Crawford C , et al. (2004) Occupational Performance History Interview II (OPHI-II) Version 2.1. Chicago, IL: Model of Occupational Therapy Clearinghouse.

[bibr21-03080226241254768] Kielhofner G Mallinson T Forsyth K , et al. (2001) Psychometric properties of the second version of the Occupational Performance History Interview (OPHI-II). American Journal of Occupational Therapy 55: 260–267. 10.5014/ajot.55.3.260 11723966

[bibr22-03080226241254768] Lee SW Morley M Taylor RR , et al. (2011) The development of care pathways and packages in mental health based on the Model of Human Occupation Screening Tool. British Journal of Occupational Therapy 74: 284–294.

[bibr23-03080226241254768] Mingus W Burchfield KB (2012) From prison to integration: Applying modified labeling theory to sex offenders. Criminal Justice Studies 25: 97–109.

[bibr24-03080226241254768] Minoudis P Kane E (2017) It’s a journey, not a destination – From dangerous and severe personality disorder (DSPD) to the offender personality disorder (OPD) pathway. Criminal behaviour and mental health 27: 207–213. 28677904 10.1002/cbm.2027

[bibr25-03080226241254768] Moore KE Stuewig JB Tangney JP (2016) The effect of stigma on criminal offenders’ functioning: A longitudinal mediational model. Deviant Behavior 37: 196–218. 26973364 10.1080/01639625.2014.1004035 PMC4788463

[bibr26-03080226241254768] NHS England (2016) Mental health clustering booklet (V5.0) (2016/2017). Available at: https://assets.publishing.service.gov.uk/government/uploads/system/uploads/attachment_data/file/499475/Annex_B4_Mental_health_clustering_booklet.pdf (accessed 4 June 2017).

[bibr27-03080226241254768] Olver M Stockdale KC Wormith JS (2014) Thirty years of research on the level of service scales: A meta-analytic examination of predictive accuracy and sources of variability. Psychological Assessment 26: 156–176. 24274046 10.1037/a0035080

[bibr28-03080226241254768] Parkinson S Forsyth K Kielhofner G (2006) The Model of Human Occupation Screening Tool (MOHOST): Version 2.0. Chicago, IL: Model of Human Occupation Clearinghouse.

[bibr29-03080226241254768] Patton MQ (2002) Qualitative Research and Evaluation Methods. Thousand Oaks, CA: SAGE Publications Inc.

[bibr30-03080226241254768] Potvin O Vallée C Larivière N , et al. (2019) Experience of occupations among people living with a personality disorder. Occupational Therapy International 2019: 1–11. 10.1155/2019/9030897 PMC6458844 31049046

[bibr31-03080226241254768] Singleton N Meltzer H Gatward R (1998) Psychiatric morbidity among prisoners in England and Wales. London: Office for National Statistics.

[bibr32-03080226241254768] Skett S (2015) Offender Personality Disorder Pathway strategy 2015. London: National Offender Management Service; NHS England.

[bibr33-03080226241254768] Twinley R (Ed.) (2020) Illuminating the Dark Side of Occupation: International Perspectives From Occupational Therapy and Occupational Science, 1st edn. London: Routledge. 10.4324/9780429266256

[bibr34-03080226241254768] World Health Organization (2002) Towards a common language for functioning, disability and health: ICF. Geneva: World Health Organization.

[bibr35-03080226241254768] World Health Organization (2018) International Statistical Classification of Diseases and Related Health Problems 11th Revision (ICD-11). Geneva: World Health Organization.

[bibr36-03080226241254768] Yu R Geddes JR Fazel S (2012) Personality disorders, violence, and antisocial behavior: A systematic review and meta-regression analysis. Journal of Personality Disorders 26: 775–792. 23013345 10.1521/pedi.2012.26.5.775

[bibr37-03080226241254768] Zanarini MC Frankenburg FR Reich DB , et al. (2012) Attainment and stability of sustained symptomatic remission and recovery among patients with borderline personality disorder and axis II comparison subjects: A 16-year prospective follow-up study. American Journal of Psychiatry 169: 476–483. 22737693 10.1176/appi.ajp.2011.11101550 PMC3509999

