# Supplementary material for: Assessing occupational participation among justice-involved people ‘with a personality disorder’: Quantitative assessments and their properties
Source: Br J Occup Ther. 2024 Jun 3;87(10):625–35. doi: 10.1177/03080226241254768 (PMC11887891; doi:10.1177/03080226241254768)
Supplement: sj-docx-1-bjo-10.1177_03080226241254768 – Supplemental material for Assessing occupational participation among justice-involved people ‘with a personality disorder’: Quantitative assessments and their properties [file sj-docx-1-bjo-10.1177_03080226241254768.docx]

# Supplementary File

**Rationale for variable dichotomisation in within sample subgroup analysis**

1. Sex – man/woman

The factors influencing occupational participation may differ between men and women, with different challenges associated with parenting for example.

2 Age – under 35/35+

The activities and roles in which people participate and the values associated with them change with age. There is also potential for both individual (e.g. skills) and environmental (social networks) influences on occupational participation to change depending on age/stage of life. The documented ‘age-crime’ curve suggests that most offenders cease criminal activity after a peak in late adolescence. However, there is a group who continue to offend at high rates into adulthood (Moffitt, 2006)

The problematic traits of Cluster B personality disorders (antisocial and borderline personality disorder) are typically what results in screening into the OPDP. There is evidence for a symptomatic recovery from personality disorder over time. However, despite symptomatic remission for 78% of people with borderline personality disorder after eight years, only 40% achieve good social and vocational functioning (Black et al., 2015; Zanarini 2012).

Comparison by age allows consideration of different experiences of occupational participation, and whether the expected improvement in personality symptoms and offending behaviour facilitate occupational participation.

3. Ethnicity – white/UK minority ethnicity

Most people screened into the OPDP are white. There may be important differences to consider that influence occupational participation based on ethnicity, such as structural racism and direct experience of racial/cultural discrimination.

4. Offence type – violent/sexual

Barriers and opportunities to occupational participation may be influenced by differences linked to different offence types, and society’s reaction to them. For example, barriers to occupational participation for sexual offenders can be high due to societal attitudes and shame.

5. ‘Successful’ occupational participation – employed/unemployed.

Employment is not the only indication of participation. However, it is the most often reported in the literature, is a valued element of occupational participation in the UK, is used as a health indicator and is associated with avoidance of further crime. It was adopted as a proxy for successful occupational participation.

**Table S1. Mean MOHOST subscale scores in study and comparison samples**

| MOHOST subscale | Sample mean | Standard error | 95% CI  Lower bound | 95% CI  Upper bound | UKMH mean | NPCC mean |
| --- | --- | --- | --- | --- | --- | --- |
| Motivation | 2.76 | 0.87 | 2.58 | 2.95 | 2.44 | 2.45 |
| Habituation | 2.35 | 0.13 | 2.07 | 2.63 | 2.31 | 2.42 |
| Communication and interaction | 2.68 | 0.16 | 2.35 | 3.01 | 2.90 | 3.25 |
| Process | 2.85 | 0.12 | 2.59 | 3.10 | 2.56 | 2.93 |
| Motor | 3.64 | 0.12 | 3.38 | 3.90 | 3.09 | 3.42 |
| Environment | 2.46 | 0.11 | 2.22 | 2.69 | 2.77 | 2.89 |

**Table S2. Mean OPHI-II scale scores in study and comparison samples**

| OPHI-II scale | Sample mean | Standard error | 95% CI  Lower bound | 95% CI  Upper bound | Physical disability mean | No diagnosis mean |
| --- | --- | --- | --- | --- | --- | --- |
| Identity | 42.61 | 2.17 | 38.03 | 47.19 | 58.59 | 60.30 |
| Identity (adjusted) | 49.89 | 2.79 | 44.01 | 55.77 | 58.59 | 60.30 |
| Competence | 40.33 | 2.77 | 34.47 | 46.19 | 46.94 | 50.14 |
| Competence (adjusted) | 50.22 | 2.70 | 44.53 | 55.92 | 46.94 | 50.14 |
| Settings | 47.44 | 2.68 | 41.79 | 53.09 | 49.59 | 55.21 |

**Table S3. Differences within the study sample by sex**

|  | Null Hypothesis | Sig.^a,b^ | Decision |
| --- | --- | --- | --- |
| 1 | The distribution of MOHOST_ApraisesAbility is the same across categories of Sex. | .924^c^ | Retain null hypothesis. |
| 2 | The distribution of MOHOST_ExpectationOfSuccess is the same across categories of Sex. | .503^c^ | Retain null hypothesis. |
| 3 | The distribution of MOHOST_Interests is the same across categories of Sex. | .924^c^ | Retain null hypothesis. |
| 4 | The distribution of MOHOST_Choices is the same across categories of Sex. | .703^c^ | Retain null hypothesis. |
| 5 | The distribution of MOHOST_Routine is the same across categories of Sex. | .566^c^ | Retain null hypothesis. |
| 6 | The distribution of MOHOST_Adaptability is the same across categories of Sex. | .775^c^ | Retain null hypothesis. |
| 7 | The distribution of MOHOST_Roles is the same across categories of Sex. | .703^c^ | Retain null hypothesis. |
| 8 | The distribution of MOHOST_Responsibility is the same across categories of Sex. | .633^c^ | Retain null hypothesis. |
| 9 | The distribution of MOHOST_NonVerbalSkills is the same across categories of Sex. | .014^c^ | Reject null hypothesis. |
| 10 | The distribution of MOHOST_Conversation is the same across categories of Sex. | .143^c^ | Retain null hypothesis. |
| 11 | The distribution of MOHOST_VocalExpression is the same across categories of Sex. | .566^c^ | Retain null hypothesis. |
| 12 | The distribution of MOHOST_Relationships is the same across categories of Sex. | .387^c^ | Retain null hypothesis. |
| 13 | The distribution of MOHOST_Knowledge is the same across categories of Sex. | .924^c^ | Retain null hypothesis. |
| 14 | The distribution of MOHOST_Timing is the same across categories of Sex. | .046^c^ | Reject null hypothesis. |
| 15 | The distribution of MOHOST_Organisation is the same across categories of Sex. | .775^c^ | Retain null hypothesis. |
| 16 | The distribution of MOHOST_ProblemSolving is the same across categories of Sex. | .924^c^ | Retain null hypothesis. |
| 17 | The distribution of MOHOST_PostureMobility is the same across categories of Sex. | .849^c^ | Retain null hypothesis. |
| 18 | The distribution of MOHOST_Coordination is the same across categories of Sex. | .336^c^ | Retain null hypothesis. |
| 19 | The distribution of MOHOST_StrengthEffort is the same across categories of Sex. | .633^c^ | Retain null hypothesis. |
| 20 | The distribution of MOHOST_Energy is the same across categories of Sex. | .924^c^ | Retain null hypothesis. |
| 21 | The distribution of MOHOST_PhysSpace is the same across categories of Sex. | .443^c^ | Retain null hypothesis. |
| 22 | The distribution of MOHOST_PhysResources is the same across categories of Sex. | .566^c^ | Retain null hypothesis. |
| 23 | The distribution of MOHOST_SocialGroups is the same across categories of Sex. | .775^c^ | Retain null hypothesis. |
| 24 | The distribution of MOHOST_OccDemands is the same across categories of Sex. | .566^c^ | Retain null hypothesis. |
| 25 | The distribution of MOHOSTSS_Motivation is the same across categories of Sex. | .443^c^ | Retain null hypothesis. |
| 26 | The distribution of MOHOSTSS_Pattern is the same across categories of Sex. | .503^c^ | Retain null hypothesis. |
| 27 | The distribution of MOHOSTSS_CommInt is the same across categories of Sex. | .387^c^ | Retain null hypothesis. |
| 28 | The distribution of MOHOSTSS_Process is the same across categories of Sex. | .566^c^ | Retain null hypothesis. |
| 29 | The distribution of MOHOSTSS_Motor is the same across categories of Sex. | .924^c^ | Retain null hypothesis. |
| 30 | The distribution of MOHOSTSS_Environment is the same across categories of Sex. | .246^c^ | Retain null hypothesis. |
| 31 | The distribution of OPHISCALE_Identity is the same across categories of Sex. | .703^c^ | Retain null hypothesis. |
| 32 | The distribution of OPHISCALE_Competence is the same across categories of Sex. | .336^c^ | Retain null hypothesis. |
| 33 | The distribution of OPHISCALE_Settings is the same across categories of Sex. | 1.000^c^ | Retain null hypothesis. |
| 34 | The distribution of OPHISCALE_ADJIdentity is the same across categories of Sex. | .443^c^ | Retain null hypothesis. |
| 35 | The distribution of OPHISCALE_ADJCompetence is the same across categories of Sex. | .849^c^ | Retain null hypothesis. |
| 36 | The distribution of OPHIIdentity_PersonalGoalsProjects is the same across categories of Sex. | .095^c^ | Retain null hypothesis. |
| 37 | The distribution of OPHIIdentity_DesiredOccLifestyle is the same across categories of Sex. | .703^c^ | Retain null hypothesis. |
| 38 | The distribution of OPHIIdentity_ExpectsSuccess is the same across categories of Sex. | .387^c^ | Retain null hypothesis. |
| 39 | The distribution of OPHIIdentity_AcceptsResponsibility is the same across categories of Sex. | .703^c^ | Retain null hypothesis. |
| 40 | The distribution of OPHIIdentity_AppraisesAbilityLimitations is the same across categories of Sex. | .703^c^ | Retain null hypothesis. |
| 41 | The distribution of OPHIIdentity_CommitmentsValues is the same across categories of Sex. | .503^c^ | Retain null hypothesis. |
| 42 | The distribution of OPHIIdentity_IdentityObligations is the same across categories of Sex. | .775^c^ | Retain null hypothesis. |
| 43 | The distribution of OPHIIdentity_Interests is the same across categories of Sex. | .173^c^ | Retain null hypothesis. |
| 44 | The distribution of OPHIIdentity_PastFeltEffective is the same across categories of Sex. | .046^c^ | Reject null hypothesis. |
| 45 | The distribution of OPHIIdentity_PastMeaningSatisfaction is the same across categories of Sex. | .173^c^ | Retain null hypothesis. |
| 46 | The distribution of OPHIIdentity_PastOccChoices is the same across categories of Sex. | .503^c^ | Retain null hypothesis. |
| 47 | The distribution of OPHIICompetence_SatisfyingLifeStyle is the same across categories of Sex. | .849^c^ | Retain null hypothesis. |
| 48 | The distribution of OPHIICompetence_RoleExpectations is the same across categories of Sex. | .703^c^ | Retain null hypothesis. |
| 49 | The distribution of OPHIICompetence_WorksTowardGoals is the same across categories of Sex. | .633^c^ | Retain null hypothesis. |
| 50 | The distribution of OPHIICompetence_PerformanceStandards is the same across categories of Sex. | .633^c^ | Retain null hypothesis. |
| 51 | The distribution of OPHIICompetence_OrganisesTime is the same across categories of Sex. | .924^c^ | Retain null hypothesis. |
| 52 | The distribution of OPHIICompetence_ParticipatesInterests is the same across categories of Sex. | .246^c^ | Retain null hypothesis. |
| 53 | The distribution of OPHIICompetence_PastRoles is the same across categories of Sex. | .566^c^ | Retain null hypothesis. |
| 54 | The distribution of OPHIICompetence_PastHabits is the same across categories of Sex. | .924^c^ | Retain null hypothesis. |
| 55 | The distribution of OPHIICompetence_PastAchievedSuccess is the same across categories of Sex. | .246^c^ | Retain null hypothesis. |
| 56 | The distribution of OPHIISettings_HomeOccForms is the same across categories of Sex. | .208^c^ | Retain null hypothesis. |
| 57 | The distribution of OPHIISettings_ProductiveRoleOccForms is the same across categories of Sex. | .633^c^ | Retain null hypothesis. |
| 58 | The distribution of OPHIISettings_LeisureOccForms is the same across categories of Sex. | .117^c^ | Retain null hypothesis. |
| 59 | The distribution of OPHIISettings_HomeSocialGroups is the same across categories of Sex. | 1.000^c^ | Retain null hypothesis. |
| 60 | The distribution of OPHIISettings_ProductiveRoleSocialGroups is the same across categories of Sex. | 1.000^c^ | Retain null hypothesis. |
| 61 | The distribution of OPHIISettings_LeisureSocialGroups is the same across categories of Sex. | 1.000^c^ | Retain null hypothesis. |
| 62 | The distribution of OPHIISettings_HomeSpaceObjectsResources is the same across categories of Sex. | .143^c^ | Retain null hypothesis. |
| 63 | The distribution of OPHIISettings_ProductiveRoleSOR is the same across categories of Sex. | .566^c^ | Retain null hypothesis. |
| 64 | The distribution of OPHIISettings_LeisureSOR is the same across categories of Sex. | .849^c^ | Retain null hypothesis. |

a significance level is 0.050

b. asymptotic difference is displayed

c. exact significance is displayed

**Table S4. Differences within the study sample by age**

|  | Null Hypothesis | Sig.^a,b^ | Decision |
| --- | --- | --- | --- |
| 1 | The distribution of MOHOST_ApraisesAbility is the same across categories of Age. | 1.000^c^ | Retain null hypothesis. |
| 2 | The distribution of MOHOST_ExpectationOfSuccess is the same across categories of Age. | .860^c^ | Retain null hypothesis. |
| 3 | The distribution of MOHOST_Interests is the same across categories of Age. | .375^c^ | Retain null hypothesis. |
| 4 | The distribution of MOHOST_Choices is the same across categories of Age. | .659^c^ | Retain null hypothesis. |
| 5 | The distribution of MOHOST_Routine is the same across categories of Age. | .860^c^ | Retain null hypothesis. |
| 6 | The distribution of MOHOST_Adaptability is the same across categories of Age. | .860^c^ | Retain null hypothesis. |
| 7 | The distribution of MOHOST_Roles is the same across categories of Age. | .425^c^ | Retain null hypothesis. |
| 8 | The distribution of MOHOST_Responsibility is the same across categories of Age. | .328^c^ | Retain null hypothesis. |
| 9 | The distribution of MOHOST_NonVerbalSkills is the same across categories of Age. | .479^c^ | Retain null hypothesis. |
| 10 | The distribution of MOHOST_Conversation is the same across categories of Age. | .930^c^ | Retain null hypothesis. |
| 11 | The distribution of MOHOST_VocalExpression is the same across categories of Age. | .425^c^ | Retain null hypothesis. |
| 12 | The distribution of MOHOST_Relationships is the same across categories of Age. | .860^c^ | Retain null hypothesis. |
| 13 | The distribution of MOHOST_Knowledge is the same across categories of Age. | .375^c^ | Retain null hypothesis. |
| 14 | The distribution of MOHOST_Timing is the same across categories of Age. | .328^c^ | Retain null hypothesis. |
| 15 | The distribution of MOHOST_Organisation is the same across categories of Age. | .930^c^ | Retain null hypothesis. |
| 16 | The distribution of MOHOST_ProblemSolving is the same across categories of Age. | .375^c^ | Retain null hypothesis. |
| 17 | The distribution of MOHOST_PostureMobility is the same across categories of Age. | .930^c^ | Retain null hypothesis. |
| 18 | The distribution of MOHOST_Coordination is the same across categories of Age. | .211^c^ | Retain null hypothesis. |
| 19 | The distribution of MOHOST_StrengthEffort is the same across categories of Age. | .328^c^ | Retain null hypothesis. |
| 20 | The distribution of MOHOST_Energy is the same across categories of Age. | 1.000^c^ | Retain null hypothesis. |
| 21 | The distribution of MOHOST_PhysSpace is the same across categories of Age. | .724^c^ | Retain null hypothesis. |
| 22 | The distribution of MOHOST_PhysResources is the same across categories of Age. | .246^c^ | Retain null hypothesis. |
| 23 | The distribution of MOHOST_SocialGroups is the same across categories of Age. | .425^c^ | Retain null hypothesis. |
| 24 | The distribution of MOHOST_OccDemands is the same across categories of Age. | .724^c^ | Retain null hypothesis. |
| 25 | The distribution of MOHOSTSS_Motivation is the same across categories of Age. | 1.000^c^ | Retain null hypothesis. |
| 26 | The distribution of MOHOSTSS_Pattern is the same across categories of Age. | .930^c^ | Retain null hypothesis. |
| 27 | The distribution of MOHOSTSS_CommInt is the same across categories of Age. | .930^c^ | Retain null hypothesis. |
| 28 | The distribution of MOHOSTSS_Process is the same across categories of Age. | .659^c^ | Retain null hypothesis. |
| 29 | The distribution of MOHOSTSS_Motor is the same across categories of Age. | .791^c^ | Retain null hypothesis. |
| 30 | The distribution of MOHOSTSS_Environment is the same across categories of Age. | .791^c^ | Retain null hypothesis. |
| 31 | The distribution of OPHISCALE_Identity is the same across categories of Age. | .536^c^ | Retain null hypothesis. |
| 32 | The distribution of OPHISCALE_Competence is the same across categories of Age. | .536^c^ | Retain null hypothesis. |
| 33 | The distribution of OPHISCALE_Settings is the same across categories of Age. | 1.000^c^ | Retain null hypothesis. |
| 34 | The distribution of OPHISCALE_ADJIdentity is the same across categories of Age. | 1.000^c^ | Retain null hypothesis. |
| 35 | The distribution of OPHISCALE_ADJCompetence is the same across categories of Age. | .659^c^ | Retain null hypothesis. |
| 36 | The distribution of OPHIIdentity_PersonalGoalsProjects is the same across categories of Age. | .596^c^ | Retain null hypothesis. |
| 37 | The distribution of OPHIIdentity_DesiredOccLifestyle is the same across categories of Age. | .179^c^ | Retain null hypothesis. |
| 38 | The distribution of OPHIIdentity_ExpectsSuccess is the same across categories of Age. | .659^c^ | Retain null hypothesis. |
| 39 | The distribution of OPHIIdentity_AcceptsResponsibility is the same across categories of Age. | .724^c^ | Retain null hypothesis. |
| 40 | The distribution of OPHIIdentity_AppraisesAbilityLimitations is the same across categories of Age. | .246^c^ | Retain null hypothesis. |
| 41 | The distribution of OPHIIdentity_CommitmentsValues is the same across categories of Age. | .425^c^ | Retain null hypothesis. |
| 42 | The distribution of OPHIIdentity_IdentityObligations is the same across categories of Age. | .930^c^ | Retain null hypothesis. |
| 43 | The distribution of OPHIIdentity_Interests is the same across categories of Age. | .246^c^ | Retain null hypothesis. |
| 44 | The distribution of OPHIIdentity_PastFeltEffective is the same across categories of Age. | .596^c^ | Retain null hypothesis. |
| 45 | The distribution of OPHIIdentity_PastMeaningSatisfaction is the same across categories of Age. | .126^c^ | Retain null hypothesis. |
| 46 | The distribution of OPHIIdentity_PastOccChoices is the same across categories of Age. | .328^c^ | Retain null hypothesis. |
| 47 | The distribution of OPHIICompetence_SatisfyingLifeStyle is the same across categories of Age. | .425^c^ | Retain null hypothesis. |
| 48 | The distribution of OPHIICompetence_RoleExpectations is the same across categories of Age. | .930^c^ | Retain null hypothesis. |
| 49 | The distribution of OPHIICompetence_WorksTowardGoals is the same across categories of Age. | .930^c^ | Retain null hypothesis. |
| 50 | The distribution of OPHIICompetence_PerformanceStandards is the same across categories of Age. | .536^c^ | Retain null hypothesis. |
| 51 | The distribution of OPHIICompetence_OrganisesTime is the same across categories of Age. | .791^c^ | Retain null hypothesis. |
| 52 | The distribution of OPHIICompetence_ParticipatesInterests is the same across categories of Age. | .375^c^ | Retain null hypothesis. |
| 53 | The distribution of OPHIICompetence_PastRoles is the same across categories of Age. | .246^c^ | Retain null hypothesis. |
| 54 | The distribution of OPHIICompetence_PastHabits is the same across categories of Age. | .246^c^ | Retain null hypothesis. |
| 55 | The distribution of OPHIICompetence_PastAchievedSuccess is the same across categories of Age. | .479^c^ | Retain null hypothesis. |
| 56 | The distribution of OPHIISettings_HomeOccForms is the same across categories of Age. | .596^c^ | Retain null hypothesis. |
| 57 | The distribution of OPHIISettings_ProductiveRoleOccForms is the same across categories of Age. | .659^c^ | Retain null hypothesis. |
| 58 | The distribution of OPHIISettings_LeisureOccForms is the same across categories of Age. | .860^c^ | Retain null hypothesis. |
| 59 | The distribution of OPHIISettings_HomeSocialGroups is the same across categories of Age. | .659^c^ | Retain null hypothesis. |
| 60 | The distribution of OPHIISettings_ProductiveRoleSocialGroups is the same across categories of Age. | .536^c^ | Retain null hypothesis. |
| 61 | The distribution of OPHIISettings_LeisureSocialGroups is the same across categories of Age. | .724^c^ | Retain null hypothesis. |
| 62 | The distribution of OPHIISettings_HomeSpaceObjectsResources is the same across categories of Age. | .930^c^ | Retain null hypothesis. |
| 63 | The distribution of OPHIISettings_ProductiveRoleSOR is the same across categories of Age. | .375^c^ | Retain null hypothesis. |
| 64 | The distribution of OPHIISettings_LeisureSOR is the same across categories of Age. | .724^c^ | Retain null hypothesis. |

a significance level is 0.050

b. asymptotic difference is displayed

c. exact significance is displayed

**Table S5. Differences within the study sample by ethnicity**

|  | Null Hypothesis | Sig.^a,b^ | Decision |
| --- | --- | --- | --- |
| 1 | The distribution of MOHOST_ApraisesAbility is the same across categories of Ethnicity. | .387^c^ | Retain null hypothesis. |
| 2 | The distribution of MOHOST_ExpectationOfSuccess is the same across categories of Ethnicity. | .503^c^ | Retain null hypothesis. |
| 3 | The distribution of MOHOST_Interests is the same across categories of Ethnicity. | .703^c^ | Retain null hypothesis. |
| 4 | The distribution of MOHOST_Choices is the same across categories of Ethnicity. | .246^c^ | Retain null hypothesis. |
| 5 | The distribution of MOHOST_Routine is the same across categories of Ethnicity. | .046^c^ | Reject null hypothesis. |
| 6 | The distribution of MOHOST_Adaptability is the same across categories of Ethnicity. | .026^c^ | Reject null hypothesis. |
| 7 | The distribution of MOHOST_Roles is the same across categories of Ethnicity. | .208^c^ | Retain null hypothesis. |
| 8 | The distribution of MOHOST_Responsibility is the same across categories of Ethnicity. | .246^c^ | Retain null hypothesis. |
| 9 | The distribution of MOHOST_NonVerbalSkills is the same across categories of Ethnicity. | .059^c^ | Retain null hypothesis. |
| 10 | The distribution of MOHOST_Conversation is the same across categories of Ethnicity. | .143^c^ | Retain null hypothesis. |
| 11 | The distribution of MOHOST_VocalExpression is the same across categories of Ethnicity. | .387^c^ | Retain null hypothesis. |
| 12 | The distribution of MOHOST_Relationships is the same across categories of Ethnicity. | .095^c^ | Retain null hypothesis. |
| 13 | The distribution of MOHOST_Knowledge is the same across categories of Ethnicity. | .566^c^ | Retain null hypothesis. |
| 14 | The distribution of MOHOST_Timing is the same across categories of Ethnicity. | .566^c^ | Retain null hypothesis. |
| 15 | The distribution of MOHOST_Organisation is the same across categories of Ethnicity. | .633^c^ | Retain null hypothesis. |
| 16 | The distribution of MOHOST_ProblemSolving is the same across categories of Ethnicity. | .075^c^ | Retain null hypothesis. |
| 17 | The distribution of MOHOST_PostureMobility is the same across categories of Ethnicity. | 1.000^c^ | Retain null hypothesis. |
| 18 | The distribution of MOHOST_Coordination is the same across categories of Ethnicity. | .849^c^ | Retain null hypothesis. |
| 19 | The distribution of MOHOST_StrengthEffort is the same across categories of Ethnicity. | .633^c^ | Retain null hypothesis. |
| 20 | The distribution of MOHOST_Energy is the same across categories of Ethnicity. | .849^c^ | Retain null hypothesis. |
| 21 | The distribution of MOHOST_PhysSpace is the same across categories of Ethnicity. | .173^c^ | Retain null hypothesis. |
| 22 | The distribution of MOHOST_PhysResources is the same across categories of Ethnicity. | .208^c^ | Retain null hypothesis. |
| 23 | The distribution of MOHOST_SocialGroups is the same across categories of Ethnicity. | .703^c^ | Retain null hypothesis. |
| 24 | The distribution of MOHOST_OccDemands is the same across categories of Ethnicity. | .246^c^ | Retain null hypothesis. |
| 25 | The distribution of MOHOSTSS_Motivation is the same across categories of Ethnicity. | .143^c^ | Retain null hypothesis. |
| 26 | The distribution of MOHOSTSS_Pattern is the same across categories of Ethnicity. | .014^c^ | Reject null hypothesis. |
| 27 | The distribution of MOHOSTSS_CommInt is the same across categories of Ethnicity. | .046^c^ | Reject null hypothesis. |
| 28 | The distribution of MOHOSTSS_Process is the same across categories of Ethnicity. | .246^c^ | Retain null hypothesis. |
| 29 | The distribution of MOHOSTSS_Motor is the same across categories of Ethnicity. | .775^c^ | Retain null hypothesis. |
| 30 | The distribution of MOHOSTSS_Environment is the same across categories of Ethnicity. | 1.000^c^ | Retain null hypothesis. |
| 31 | The distribution of OPHISCALE_Identity is the same across categories of Ethnicity. | .095^c^ | Retain null hypothesis. |
| 32 | The distribution of OPHISCALE_Competence is the same across categories of Ethnicity. | .566^c^ | Retain null hypothesis. |
| 33 | The distribution of OPHISCALE_Settings is the same across categories of Ethnicity. | .026^c^ | Reject null hypothesis. |
| 34 | The distribution of OPHISCALE_ADJIdentity is the same across categories of Ethnicity. | .059^c^ | Retain null hypothesis. |
| 35 | The distribution of OPHISCALE_ADJCompetence is the same across categories of Ethnicity. | .117^c^ | Retain null hypothesis. |
| 36 | The distribution of OPHIIdentity_PersonalGoalsProjects is the same across categories of Ethnicity. | .019^c^ | Reject null hypothesis. |
| 37 | The distribution of OPHIIdentity_DesiredOccLifestyle is the same across categories of Ethnicity. | .336^c^ | Retain null hypothesis. |
| 38 | The distribution of OPHIIdentity_ExpectsSuccess is the same across categories of Ethnicity. | .924^c^ | Retain null hypothesis. |
| 39 | The distribution of OPHIIdentity_AcceptsResponsibility is the same across categories of Ethnicity. | .208^c^ | Retain null hypothesis. |
| 40 | The distribution of OPHIIdentity_AppraisesAbilityLimitations is the same across categories of Ethnicity. | .703^c^ | Retain null hypothesis. |
| 41 | The distribution of OPHIIdentity_CommitmentsValues is the same across categories of Ethnicity. | 1.000^c^ | Retain null hypothesis. |
| 42 | The distribution of OPHIIdentity_IdentityObligations is the same across categories of Ethnicity. | .095^c^ | Retain null hypothesis. |
| 43 | The distribution of OPHIIdentity_Interests is the same across categories of Ethnicity. | 1.000^c^ | Retain null hypothesis. |
| 44 | The distribution of OPHIIdentity_PastFeltEffective is the same across categories of Ethnicity. | .924^c^ | Retain null hypothesis. |
| 45 | The distribution of OPHIIdentity_PastMeaningSatisfaction is the same across categories of Ethnicity. | .703^c^ | Retain null hypothesis. |
| 46 | The distribution of OPHIIdentity_PastOccChoices is the same across categories of Ethnicity. | .503^c^ | Retain null hypothesis. |
| 47 | The distribution of OPHIICompetence_SatisfyingLifeStyle is the same across categories of Ethnicity. | .117^c^ | Retain null hypothesis. |
| 48 | The distribution of OPHIICompetence_RoleExpectations is the same across categories of Ethnicity. | .143^c^ | Retain null hypothesis. |
| 49 | The distribution of OPHIICompetence_WorksTowardGoals is the same across categories of Ethnicity. | .849^c^ | Retain null hypothesis. |
| 50 | The distribution of OPHIICompetence_PerformanceStandards is the same across categories of Ethnicity. | .173^c^ | Retain null hypothesis. |
| 51 | The distribution of OPHIICompetence_OrganisesTime is the same across categories of Ethnicity. | .246^c^ | Retain null hypothesis. |
| 52 | The distribution of OPHIICompetence_ParticipatesInterests is the same across categories of Ethnicity. | 1.000^c^ | Retain null hypothesis. |
| 53 | The distribution of OPHIICompetence_PastRoles is the same across categories of Ethnicity. | .775^c^ | Retain null hypothesis. |
| 54 | The distribution of OPHIICompetence_PastHabits is the same across categories of Ethnicity. | .336^c^ | Retain null hypothesis. |
| 55 | The distribution of OPHIICompetence_PastAchievedSuccess is the same across categories of Ethnicity. | .775^c^ | Retain null hypothesis. |
| 56 | The distribution of OPHIISettings_HomeOccForms is the same across categories of Ethnicity. | .503^c^ | Retain null hypothesis. |
| 57 | The distribution of OPHIISettings_ProductiveRoleOccForms is the same across categories of Ethnicity. | .035^c^ | Reject null hypothesis. |
| 58 | The distribution of OPHIISettings_LeisureOccForms is the same across categories of Ethnicity. | .289^c^ | Retain null hypothesis. |
| 59 | The distribution of OPHIISettings_HomeSocialGroups is the same across categories of Ethnicity. | .703^c^ | Retain null hypothesis. |
| 60 | The distribution of OPHIISettings_ProductiveRoleSocialGroups is the same across categories of Ethnicity. | .026^c^ | Reject null hypothesis. |
| 61 | The distribution of OPHIISettings_LeisureSocialGroups is the same across categories of Ethnicity. | .443^c^ | Retain null hypothesis. |
| 62 | The distribution of OPHIISettings_HomeSpaceObjectsResources is the same across categories of Ethnicity. | .387^c^ | Retain null hypothesis. |
| 63 | The distribution of OPHIISettings_ProductiveRoleSOR is the same across categories of Ethnicity. | .387^c^ | Retain null hypothesis. |
| 64 | The distribution of OPHIISettings_LeisureSOR is the same across categories of Ethnicity. | .026^c^ | Reject null hypothesis. |

a significance level is 0.050

b. asymptotic difference is displayed

c. exact significance is displayed

**Table S6. Differences within the study sample by offence type**

|  | Null Hypothesis | Sig.^a,b^ | Decision |
| --- | --- | --- | --- |
| 1 | The distribution of MOHOST_ApraisesAbility is the same across categories of OffenceType. | .566^c^ | Retain null hypothesis. |
| 2 | The distribution of MOHOST_ExpectationOfSuccess is the same across categories of OffenceType. | .143^c^ | Retain null hypothesis. |
| 3 | The distribution of MOHOST_Interests is the same across categories of OffenceType. | .924^c^ | Retain null hypothesis. |
| 4 | The distribution of MOHOST_Choices is the same across categories of OffenceType. | .703^c^ | Retain null hypothesis. |
| 5 | The distribution of MOHOST_Routine is the same across categories of OffenceType. | .566^c^ | Retain null hypothesis. |
| 6 | The distribution of MOHOST_Adaptability is the same across categories of OffenceType. | .924^c^ | Retain null hypothesis. |
| 7 | The distribution of MOHOST_Roles is the same across categories of OffenceType. | .246^c^ | Retain null hypothesis. |
| 8 | The distribution of MOHOST_Responsibility is the same across categories of OffenceType. | .849^c^ | Retain null hypothesis. |
| 9 | The distribution of MOHOST_NonVerbalSkills is the same across categories of OffenceType. | .703^c^ | Retain null hypothesis. |
| 10 | The distribution of MOHOST_Conversation is the same across categories of OffenceType. | .443^c^ | Retain null hypothesis. |
| 11 | The distribution of MOHOST_VocalExpression is the same across categories of OffenceType. | .566^c^ | Retain null hypothesis. |
| 12 | The distribution of MOHOST_Relationships is the same across categories of OffenceType. | .924^c^ | Retain null hypothesis. |
| 13 | The distribution of MOHOST_Knowledge is the same across categories of OffenceType. | .289^c^ | Retain null hypothesis. |
| 14 | The distribution of MOHOST_Timing is the same across categories of OffenceType. | .387^c^ | Retain null hypothesis. |
| 15 | The distribution of MOHOST_Organisation is the same across categories of OffenceType. | 1.000^c^ | Retain null hypothesis. |
| 16 | The distribution of MOHOST_ProblemSolving is the same across categories of OffenceType. | .924^c^ | Retain null hypothesis. |
| 17 | The distribution of MOHOST_PostureMobility is the same across categories of OffenceType. | .503^c^ | Retain null hypothesis. |
| 18 | The distribution of MOHOST_Coordination is the same across categories of OffenceType. | .336^c^ | Retain null hypothesis. |
| 19 | The distribution of MOHOST_StrengthEffort is the same across categories of OffenceType. | .633^c^ | Retain null hypothesis. |
| 20 | The distribution of MOHOST_Energy is the same across categories of OffenceType. | .246^c^ | Retain null hypothesis. |
| 21 | The distribution of MOHOST_PhysSpace is the same across categories of OffenceType. | .566^c^ | Retain null hypothesis. |
| 22 | The distribution of MOHOST_PhysResources is the same across categories of OffenceType. | 1.000^c^ | Retain null hypothesis. |
| 23 | The distribution of MOHOST_SocialGroups is the same across categories of OffenceType. | .775^c^ | Retain null hypothesis. |
| 24 | The distribution of MOHOST_OccDemands is the same across categories of OffenceType. | .117^c^ | Retain null hypothesis. |
| 25 | The distribution of MOHOSTSS_Motivation is the same across categories of OffenceType. | .443^c^ | Retain null hypothesis. |
| 26 | The distribution of MOHOSTSS_Pattern is the same across categories of OffenceType. | .503^c^ | Retain null hypothesis. |
| 27 | The distribution of MOHOSTSS_CommInt is the same across categories of OffenceType. | .703^c^ | Retain null hypothesis. |
| 28 | The distribution of MOHOSTSS_Process is the same across categories of OffenceType. | 1.000^c^ | Retain null hypothesis. |
| 29 | The distribution of MOHOSTSS_Motor is the same across categories of OffenceType. | .075^c^ | Retain null hypothesis. |
| 30 | The distribution of MOHOSTSS_Environment is the same across categories of OffenceType. | .246^c^ | Retain null hypothesis. |
| 31 | The distribution of OPHISCALE_Identity is the same across categories of OffenceType. | .443^c^ | Retain null hypothesis. |
| 32 | The distribution of OPHISCALE_Competence is the same across categories of OffenceType. | .503^c^ | Retain null hypothesis. |
| 33 | The distribution of OPHISCALE_Settings is the same across categories of OffenceType. | .336^c^ | Retain null hypothesis. |
| 34 | The distribution of OPHISCALE_ADJIdentity is the same across categories of OffenceType. | .503^c^ | Retain null hypothesis. |
| 35 | The distribution of OPHISCALE_ADJCompetence is the same across categories of OffenceType. | .775^c^ | Retain null hypothesis. |
| 36 | The distribution of OPHIIdentity_PersonalGoalsProjects is the same across categories of OffenceType. | .289^c^ | Retain null hypothesis. |
| 37 | The distribution of OPHIIdentity_DesiredOccLifestyle is the same across categories of OffenceType. | .849^c^ | Retain null hypothesis. |
| 38 | The distribution of OPHIIdentity_ExpectsSuccess is the same across categories of OffenceType. | .387^c^ | Retain null hypothesis. |
| 39 | The distribution of OPHIIdentity_AcceptsResponsibility is the same across categories of OffenceType. | .703^c^ | Retain null hypothesis. |
| 40 | The distribution of OPHIIdentity_AppraisesAbilityLimitations is the same across categories of OffenceType. | .703^c^ | Retain null hypothesis. |
| 41 | The distribution of OPHIIdentity_CommitmentsValues is the same across categories of OffenceType. | .503^c^ | Retain null hypothesis. |
| 42 | The distribution of OPHIIdentity_IdentityObligations is the same across categories of OffenceType. | .075^c^ | Retain null hypothesis. |
| 43 | The distribution of OPHIIdentity_Interests is the same across categories of OffenceType. | 1.000^c^ | Retain null hypothesis. |
| 44 | The distribution of OPHIIdentity_PastFeltEffective is the same across categories of OffenceType. | .775^c^ | Retain null hypothesis. |
| 45 | The distribution of OPHIIdentity_PastMeaningSatisfaction is the same across categories of OffenceType. | .775^c^ | Retain null hypothesis. |
| 46 | The distribution of OPHIIdentity_PastOccChoices is the same across categories of OffenceType. | .924^c^ | Retain null hypothesis. |
| 47 | The distribution of OPHIICompetence_SatisfyingLifeStyle is the same across categories of OffenceType. | 1.000^c^ | Retain null hypothesis. |
| 48 | The distribution of OPHIICompetence_RoleExpectations is the same across categories of OffenceType. | .924^c^ | Retain null hypothesis. |
| 49 | The distribution of OPHIICompetence_WorksTowardGoals is the same across categories of OffenceType. | .633^c^ | Retain null hypothesis. |
| 50 | The distribution of OPHIICompetence_PerformanceStandards is the same across categories of OffenceType. | .117^c^ | Retain null hypothesis. |
| 51 | The distribution of OPHIICompetence_OrganisesTime is the same across categories of OffenceType. | .703^c^ | Retain null hypothesis. |
| 52 | The distribution of OPHIICompetence_ParticipatesInterests is the same across categories of OffenceType. | .849^c^ | Retain null hypothesis. |
| 53 | The distribution of OPHIICompetence_PastRoles is the same across categories of OffenceType. | .849^c^ | Retain null hypothesis. |
| 54 | The distribution of OPHIICompetence_PastHabits is the same across categories of OffenceType. | .503^c^ | Retain null hypothesis. |
| 55 | The distribution of OPHIICompetence_PastAchievedSuccess is the same across categories of OffenceType. | .566^c^ | Retain null hypothesis. |
| 56 | The distribution of OPHIISettings_HomeOccForms is the same across categories of OffenceType. | .246^c^ | Retain null hypothesis. |
| 57 | The distribution of OPHIISettings_ProductiveRoleOccForms is the same across categories of OffenceType. | .503^c^ | Retain null hypothesis. |
| 58 | The distribution of OPHIISettings_LeisureOccForms is the same across categories of OffenceType. | .336^c^ | Retain null hypothesis. |
| 59 | The distribution of OPHIISettings_HomeSocialGroups is the same across categories of OffenceType. | .775^c^ | Retain null hypothesis. |
| 60 | The distribution of OPHIISettings_ProductiveRoleSocialGroups is the same across categories of OffenceType. | .633^c^ | Retain null hypothesis. |
| 61 | The distribution of OPHIISettings_LeisureSocialGroups is the same across categories of OffenceType. | 1.000^c^ | Retain null hypothesis. |
| 62 | The distribution of OPHIISettings_HomeSpaceObjectsResources is the same across categories of OffenceType. | 1.000^c^ | Retain null hypothesis. |
| 63 | The distribution of OPHIISettings_ProductiveRoleSOR is the same across categories of OffenceType. | .443^c^ | Retain null hypothesis. |
| 64 | The distribution of OPHIISettings_LeisureSOR is the same across categories of OffenceType. | .336^c^ | Retain null hypothesis. |

a significance level is 0.050

b. asymptotic difference is displayed

c. exact significance is displayed

**Table S7. Differences within the study sample by employment status**

|  | Null Hypothesis | Sig.^a,b^ | Decision |
| --- | --- | --- | --- |
| 1 | The distribution of MOHOST_ApraisesAbility is the same across categories of EmploymentStatus. | .849^c^ | Retain null hypothesis. |
| 2 | The distribution of MOHOST_ExpectationOfSuccess is the same across categories of EmploymentStatus. | .143^c^ | Retain null hypothesis. |
| 3 | The distribution of MOHOST_Interests is the same across categories of EmploymentStatus. | .924^c^ | Retain null hypothesis. |
| 4 | The distribution of MOHOST_Choices is the same across categories of EmploymentStatus. | .143^c^ | Retain null hypothesis. |
| 5 | The distribution of MOHOST_Routine is the same across categories of EmploymentStatus. | .075^c^ | Retain null hypothesis. |
| 6 | The distribution of MOHOST_Adaptability is the same across categories of EmploymentStatus. | .246^c^ | Retain null hypothesis. |
| 7 | The distribution of MOHOST_Roles is the same across categories of EmploymentStatus. | .503^c^ | Retain null hypothesis. |
| 8 | The distribution of MOHOST_Responsibility is the same across categories of EmploymentStatus. | .046^c^ | Reject null hypothesis. |
| 9 | The distribution of MOHOST_NonVerbalSkills is the same across categories of EmploymentStatus. | .924^c^ | Retain null hypothesis. |
| 10 | The distribution of MOHOST_Conversation is the same across categories of EmploymentStatus. | 1.000^c^ | Retain null hypothesis. |
| 11 | The distribution of MOHOST_VocalExpression is the same across categories of EmploymentStatus. | .703^c^ | Retain null hypothesis. |
| 12 | The distribution of MOHOST_Relationships is the same across categories of EmploymentStatus. | .246^c^ | Retain null hypothesis. |
| 13 | The distribution of MOHOST_Knowledge is the same across categories of EmploymentStatus. | .075^c^ | Retain null hypothesis. |
| 14 | The distribution of MOHOST_Timing is the same across categories of EmploymentStatus. | .924^c^ | Retain null hypothesis. |
| 15 | The distribution of MOHOST_Organisation is the same across categories of EmploymentStatus. | 1.000^c^ | Retain null hypothesis. |
| 16 | The distribution of MOHOST_ProblemSolving is the same across categories of EmploymentStatus. | .046^c^ | Reject null hypothesis. |
| 17 | The distribution of MOHOST_PostureMobility is the same across categories of EmploymentStatus. | 1.000^c^ | Retain null hypothesis. |
| 18 | The distribution of MOHOST_Coordination is the same across categories of EmploymentStatus. | .566^c^ | Retain null hypothesis. |
| 19 | The distribution of MOHOST_StrengthEffort is the same across categories of EmploymentStatus. | .633^c^ | Retain null hypothesis. |
| 20 | The distribution of MOHOST_Energy is the same across categories of EmploymentStatus. | .387^c^ | Retain null hypothesis. |
| 21 | The distribution of MOHOST_PhysSpace is the same across categories of EmploymentStatus. | 1.000^c^ | Retain null hypothesis. |
| 22 | The distribution of MOHOST_PhysResources is the same across categories of EmploymentStatus. | 1.000^c^ | Retain null hypothesis. |
| 23 | The distribution of MOHOST_SocialGroups is the same across categories of EmploymentStatus. | .775^c^ | Retain null hypothesis. |
| 24 | The distribution of MOHOST_OccDemands is the same across categories of EmploymentStatus. | .246^c^ | Retain null hypothesis. |
| 25 | The distribution of MOHOSTSS_Motivation is the same across categories of EmploymentStatus. | .143^c^ | Retain null hypothesis. |
| 26 | The distribution of MOHOSTSS_Pattern is the same across categories of EmploymentStatus. | .046^c^ | Reject null hypothesis. |
| 27 | The distribution of MOHOSTSS_CommInt is the same across categories of EmploymentStatus. | .703^c^ | Retain null hypothesis. |
| 28 | The distribution of MOHOSTSS_Process is the same across categories of EmploymentStatus. | .117^c^ | Retain null hypothesis. |
| 29 | The distribution of MOHOSTSS_Motor is the same across categories of EmploymentStatus. | .566^c^ | Retain null hypothesis. |
| 30 | The distribution of MOHOSTSS_Environment is the same across categories of EmploymentStatus. | .775^c^ | Retain null hypothesis. |
| 31 | The distribution of OPHISCALE_Identity is the same across categories of EmploymentStatus. | .003^c^ | Reject null hypothesis. |
| 32 | The distribution of OPHISCALE_Competence is the same across categories of EmploymentStatus. | <.001^c^ | Reject null hypothesis. |
| 33 | The distribution of OPHISCALE_Settings is the same across categories of EmploymentStatus. | .075^c^ | Retain null hypothesis. |
| 34 | The distribution of OPHISCALE_ADJIdentity is the same across categories of EmploymentStatus. | .007^c^ | Reject null hypothesis. |
| 35 | The distribution of OPHISCALE_ADJCompetence is the same across categories of EmploymentStatus. | .002^c^ | Reject null hypothesis. |
| 36 | The distribution of OPHIIdentity_PersonalGoalsProjects is the same across categories of EmploymentStatus. | .019^c^ | Reject null hypothesis. |
| 37 | The distribution of OPHIIdentity_DesiredOccLifestyle is the same across categories of EmploymentStatus. | .075^c^ | Retain null hypothesis. |
| 38 | The distribution of OPHIIdentity_ExpectsSuccess is the same across categories of EmploymentStatus. | .095^c^ | Retain null hypothesis. |
| 39 | The distribution of OPHIIdentity_AcceptsResponsibility is the same across categories of EmploymentStatus. | .703^c^ | Retain null hypothesis. |
| 40 | The distribution of OPHIIdentity_AppraisesAbilityLimitations is the same across categories of EmploymentStatus. | .703^c^ | Retain null hypothesis. |
| 41 | The distribution of OPHIIdentity_CommitmentsValues is the same across categories of EmploymentStatus. | .246^c^ | Retain null hypothesis. |
| 42 | The distribution of OPHIIdentity_IdentityObligations is the same across categories of EmploymentStatus. | .336^c^ | Retain null hypothesis. |
| 43 | The distribution of OPHIIdentity_Interests is the same across categories of EmploymentStatus. | .443^c^ | Retain null hypothesis. |
| 44 | The distribution of OPHIIdentity_PastFeltEffective is the same across categories of EmploymentStatus. | .075^c^ | Retain null hypothesis. |
| 45 | The distribution of OPHIIdentity_PastMeaningSatisfaction is the same across categories of EmploymentStatus. | .117^c^ | Retain null hypothesis. |
| 46 | The distribution of OPHIIdentity_PastOccChoices is the same across categories of EmploymentStatus. | .019^c^ | Reject null hypothesis. |
| 47 | The distribution of OPHIICompetence_SatisfyingLifeStyle is the same across categories of EmploymentStatus. | .014^c^ | Reject null hypothesis. |
| 48 | The distribution of OPHIICompetence_RoleExpectations is the same across categories of EmploymentStatus. | .026^c^ | Reject null hypothesis. |
| 49 | The distribution of OPHIICompetence_WorksTowardGoals is the same across categories of EmploymentStatus. | .026^c^ | Reject null hypothesis. |
| 50 | The distribution of OPHIICompetence_PerformanceStandards is the same across categories of EmploymentStatus. | .173^c^ | Retain null hypothesis. |
| 51 | The distribution of OPHIICompetence_OrganisesTime is the same across categories of EmploymentStatus. | .003^c^ | Reject null hypothesis. |
| 52 | The distribution of OPHIICompetence_ParticipatesInterests is the same across categories of EmploymentStatus. | .035^c^ | Reject null hypothesis. |
| 53 | The distribution of OPHIICompetence_PastRoles is the same across categories of EmploymentStatus. | .026^c^ | Reject null hypothesis. |
| 54 | The distribution of OPHIICompetence_PastHabits is the same across categories of EmploymentStatus. | .095^c^ | Retain null hypothesis. |
| 55 | The distribution of OPHIICompetence_PastAchievedSuccess is the same across categories of EmploymentStatus. | .566^c^ | Retain null hypothesis. |
| 56 | The distribution of OPHIISettings_HomeOccForms is the same across categories of EmploymentStatus. | .059^c^ | Retain null hypothesis. |
| 57 | The distribution of OPHIISettings_ProductiveRoleOccForms is the same across categories of EmploymentStatus. | .924^c^ | Retain null hypothesis. |
| 58 | The distribution of OPHIISettings_LeisureOccForms is the same across categories of EmploymentStatus. | .703^c^ | Retain null hypothesis. |
| 59 | The distribution of OPHIISettings_HomeSocialGroups is the same across categories of EmploymentStatus. | .143^c^ | Retain null hypothesis. |
| 60 | The distribution of OPHIISettings_ProductiveRoleSocialGroups is the same across categories of EmploymentStatus. | .095^c^ | Retain null hypothesis. |
| 61 | The distribution of OPHIISettings_LeisureSocialGroups is the same across categories of EmploymentStatus. | .246^c^ | Retain null hypothesis. |
| 62 | The distribution of OPHIISettings_HomeSpaceObjectsResources is the same across categories of EmploymentStatus. | 1.000^c^ | Retain null hypothesis. |
| 63 | The distribution of OPHIISettings_ProductiveRoleSOR is the same across categories of EmploymentStatus. | .143^c^ | Retain null hypothesis. |
| 64 | The distribution of OPHIISettings_LeisureSOR is the same across categories of EmploymentStatus. | .633^c^ | Retain null hypothesis. |

a significance level is 0.050

b. asymptotic difference is displayed

c. exact significance is displayed
